# Prevalence of a History of Metabolic or Hypertensive Pregnancy Disorder in Patients With Myocardial Infarction and Non-obstructive Coronary Arteries: An Observational Cohort Study

**DOI:** 10.3389/fcvm.2022.932799

**Published:** 2022-07-15

**Authors:** Tobias F. S. Pustjens, Nousjka P. A. Vranken, Gwyneth Jansen, Patty J. C. Winkler, Mera Stein, Loes Hoebers, Bas Kietselaer, Marc E. A. Spaanderman, Saman Rasoul, Chahinda Ghossein-Doha, Arnoud W. J. van 't Hof

**Affiliations:** ^1^Department of Cardiology, Zuyderland Medical Center, Heerlen, Netherlands; ^2^Cardiovascular Research Institute Maastricht (CARIM), Maastricht University, Maastricht, Netherlands; ^3^GROW School for Oncology and Reproduction, Maastricht, Netherlands; ^4^Department of Obstetrics and Gynecology, Maastricht University Medical Centre, Maastricht, Netherlands; ^5^Department of Cardiology, Maastricht University Medical Center, Maastricht, Netherlands

**Keywords:** MINOCA, gestational hypertension, gestational diabetes, preeclampsia, cardiovascular risk factor

## Abstract

**Introduction:**

Myocardial infarction with non-obstructive coronary arteries (MINOCA) predominantly affects younger females. Women with a history of gestational hypertension (GH), preeclampsia (PE), and gestational diabetes mellitus (GDM) are subjected to an elevated lifetime risk of cardiovascular disease. However, data on the potential association between these obstetric complications and MINOCA is lacking. Therefore, the current study aimed to provide insight in the prevalence of metabolic and hypertensive pregnancy disorders (MHPD) in MINOCA patients and their clinical characteristics.

**Methods:**

In this observational cohort study conducted at the Zuyderland Medical Center and Maastricht University Medical Center in the Netherlands, patients were enrolled if they were identified as having MINOCA. Data on individual patient characteristics, laboratory results, electrocardiography as well as (non-)invasive imaging procedures were derived from the electronic health record system. Patients were asked to complete a questionnaire about prior MHPD including GDM, GH, and PE. Patients were grouped into those with MHPD and those with prior uncomplicated normotensive pregnancy (or pregnancies; NP).

**Results:**

After excluding patients without 1-year follow-up (*n* = 53), 86 female MINOCA patients remained eligible for analysis. Of the total female population, 25 (29.1%) patients had MHPD, including GH (*n* = 19; 22.1%), PE (*n* = 4; 4.7%), and GDM (*n* = 7; 8.1%). The MHPD patients showed higher rates of chronic hypertension (84.0 vs. 55.7%; *p* = 0.013), hypercholesterolemia (64.0 vs. 34.4%; *p* = 0.012), a family history of CVD (84.0 vs. 45.9%; *p* = 0.001), gout or rheumatic arthritis (16.0 vs. 1.6%; *p* = 0.024), and were more often non-smokers (45.8 vs. 78.3%; *p* = 0.004), compared to the NP patients. Moreover, MHPD patients were more likely to use cardiovascular medications at baseline. A trend toward no specific cause found for the MINOCA event was observed in MHPD patients compared to the NP group (64.0 vs. 42.6%, *p* = 0.072).

**Conclusion:**

A history of metabolic and hypertensive pregnancy disorders occurred in one-third of female MINOCA patients. In these patients, conventional cardiovascular risk factors were more prevalent compared to NP patients. In most MHPD patients, the specific cause for MINOCA remained unclear.

## Introduction

Hemodynamic and metabolic changes during pregnancy impose substantial challenges to the maternal cardiovascular system, which can result in pregnancy-associated vascular and metabolic complications. Hypertensive pregnancy complications occur in up to 10% of pregnancies and include gestational hypertension (GH) and preeclampsia (PE). Hypertensive pregnancy complications, especially PE, relate to serious short term fetal and maternal complications. With regards to long term, hypertensive pregnancy complications have shown to be associated with a two- to seven-fold increased risk of ischemic heart disease, cerebrovascular accidents and heart failure in the two decades following childbirth (1, 2). PE is thought to be an endothelial derangement superimposed upon pre-existing circulatory, metabolic, hemostatic, or immunological abnormalities. Gestational diabetes mellitus (GDM) occurs in around six percent of pregnancies with an increasing incidence over the past few decades, in part due to increasing mean maternal age and increasing prevalence of obesity (2–6). GDM is associated with development of hypertensive disorders, type 2 diabetes, and cardiovascular disease (CVD) including coronary heart disease and acute myocardial infarction (AMI) in later stages of life (7–9).

While AMI commonly presents with obstructive coronary artery disease requiring urgent revascularization, in up to 15% of AMI patients, predominantly younger females, do not show obstructed coronary arteries on coronary angiography (10). For this clinical entity, the term “myocardial infarction with non-obstructive coronary arteries” (MINOCA) has been coined (11). The underlying pathology responsible for the MINOCA event varies widely, including coronary-related causes (e.g., plaque rupture, spasm, microvascular dysfunction, dissection), and conditions mimicking MINOCA (additional diagnostic measures revealing i.e., Takotsubo cardiomyopathy or myocarditis). Notably, the underlying condition remains unknown in a significant proportion of patients (10, 12). Although previous studies evaluated the association of obstetric complications with AMI due to epicardial coronary obstructions (13, 14), data on the potential relationship between cardiovascular and metabolic pregnancy-related complications and MINOCA is still lacking. Therefore, the current study aimed to provide insight in the prevalence of prior metabolic and hypertensive pregnancy disorders (MHPD) in MINOCA patients, and their clinical characteristics.

## Methods

### Study Design and Population

This observational cohort study was conducted at the Zuyderland Medical Center and Maastricht University Medical Center in the Netherlands. The study adhered to the Declaration of Helsinki (15), and the institutional review board of both centers approved the study the protocol (Z2018137). All patients provided written informed consent prior to study inclusion.

Patients were enrolled in the registry if they had a clinical presentation identified as MINOCA. More specifically, patients were suitable for study inclusion directly following coronary angiography if (1) the confirmed diagnosis met the Fourth Universal Definition of Myocardial Infarction (16), (2) the coronary angiography performed during the index hospitalization revealed nonobstructive coronaries [coronary stenosis <50% in any potential infarct-related artery, and, if performed, non-significant fractional flow reserve assessment (i.e., FFR >0.80)], and (3) there was no other clinically overt specific cause for the acute presentation (Table 1). This way, MINOCA was regarded as a dynamic diagnosis requiring further evaluation. The degree of coronary stenosis was assessed by the treating interventional cardiologist. Nonobstructive coronary arteries on angiography were grouped into normal coronary arteries (no angiographic stenoses), and moderate coronary atherosclerotic lesions (stenoses <50%).

**Table 1:** Definition of MINOCA.

**Definition of MINOCA**
**1. Acute myocardial infarction (AMI) criteria** Clinical evidence of AMI including any of the following: • Symptoms — chest pain criteria • ECG — new changes including ST segments, LBBB, pathological Q waves • Myocardial perfusion imaging — new loss of viable myocardium • Left ventricular functional imaging — new regional wall motion abnormality
**2. Nonobstructive coronary arteries** No stenosis ≥50%
**3. No clinically overt cause for AMI presentation**

*LBBB, left bundle branch block; MINOCA, myocardial infarction with nonobstructive coronary arteries*.

Patients were not enrolled if they were aged <18 or >80 years, had severe renal disease at the time of presentation (defined as serum creatinine >150 μmol/L), had previously documented cardiomyopathy, or if there was an overt alternative explanation for elevated troponin levels rather than coronary-related ischemia.

After informed consent, patients completed a baseline questionnaire to evaluate the presence of cardiovascular risk factors [including conventional risk factors, obstetric risk factors (i.e., GH, PE, GDM), moderate exercise <30 min per day, and emotional stress]. In general, (1) GH is defined as a systolic blood pressure of 140 mmHg or more, or a diastolic blood pressure of 90 mmHg or more, on two occasions at least 4 h apart after 20 weeks of gestation, (2) gestational diabetes mellitus (GDM) is any form of hyperglycemia detected during pregnancy, regardless of whether this abnormality disappears after pregnancy, and (3) pre-eclampsia (PE) refers to the new onset of hypertension and proteinuria or other signs of end-organ damage after 20 weeks of gestation or postpartum in a previously normotensive patient (17–19). Patients were treated with optimal medical therapy according to current guidelines and at the discretion of the treating cardiologist. Follow-up data and events were reported at 1, 2, and 3 years following the index hospitalization. Only patients who had completed the 1-year follow-up were included in the current analysis to assure completeness of data on additional examination in an attempt to define the underlying cause of the MINOCA event. For the current study, female MINOCA patients were grouped into those with prior metabolic and hypertensive pregnancy disorders (MHPD) and those with a prior normal pregnancy (or pregnancies; NP).

### Data Collection

Data on individual patient characteristics, laboratory results, electrocardiography as well as (non-) invasive imaging procedures were derived from the electronic health record system and the self-reported questionnaire. Additional diagnostic work-up was performed at the discretion of the treating cardiologist and included non-invasive imaging [echocardiography, cardiac magnetic resonance imaging (CMR), or computed tomography angiography (CT-a)]. For CMR, the acquisition imaging protocol included cine imaging, late gadolinium enhancement (LGE) to detect focal fibrosis and T_2_ weighted imaging to detect myocardial edema. LGE was considered present if observed in multiple views and extended beyond right ventricular insertion areas.

If no or moderate coronary artery disease (coronary stenoses <50%) was present, subsequent coronary optical coherence tomography (OCT) or coronary function tests were performed at the operator's discretion during the index procedure or in a second procedure to assess coronary vasoreactivity and microvascular dysfunction. Coronary function tests consisted of epicardial or microvascular spasm testing with an intracoronary acetylcholine provocation test, or microvascular resistance testing by the index of microvascular resistance (IMR, abnormal if >25) and coronary flow reserve (CFR, abnormal if <2.0).

The final diagnosis of MINOCA was based on the conclusion resulting from thorough review of patient's hospital records (performed additional examination, and discharge and outpatient follow-up documentation) by 2 investigators (NV and TP).

### Statistical Analysis

Descriptive statistics for continuous variables were presented as mean with standard deviation (SD) or median and interquartile range [IQR], depending on data distribution. Either the unpaired Student's *T*-test or Mann–Whitney *U*-test was used to analyze differences in continuous parameters across study groups. Categorical data were reported as frequency values and assessed using Pearson's *X*^2^-test and the Fisher's exact test, where appropriate.

A two-tailed *p*-value <0.05 was considered statistically significant for all tests. Statistical analyses were performed using SPSS version 26.0 (IBM Corp, New York, NY, USA).

## Results

**Figure 1:**
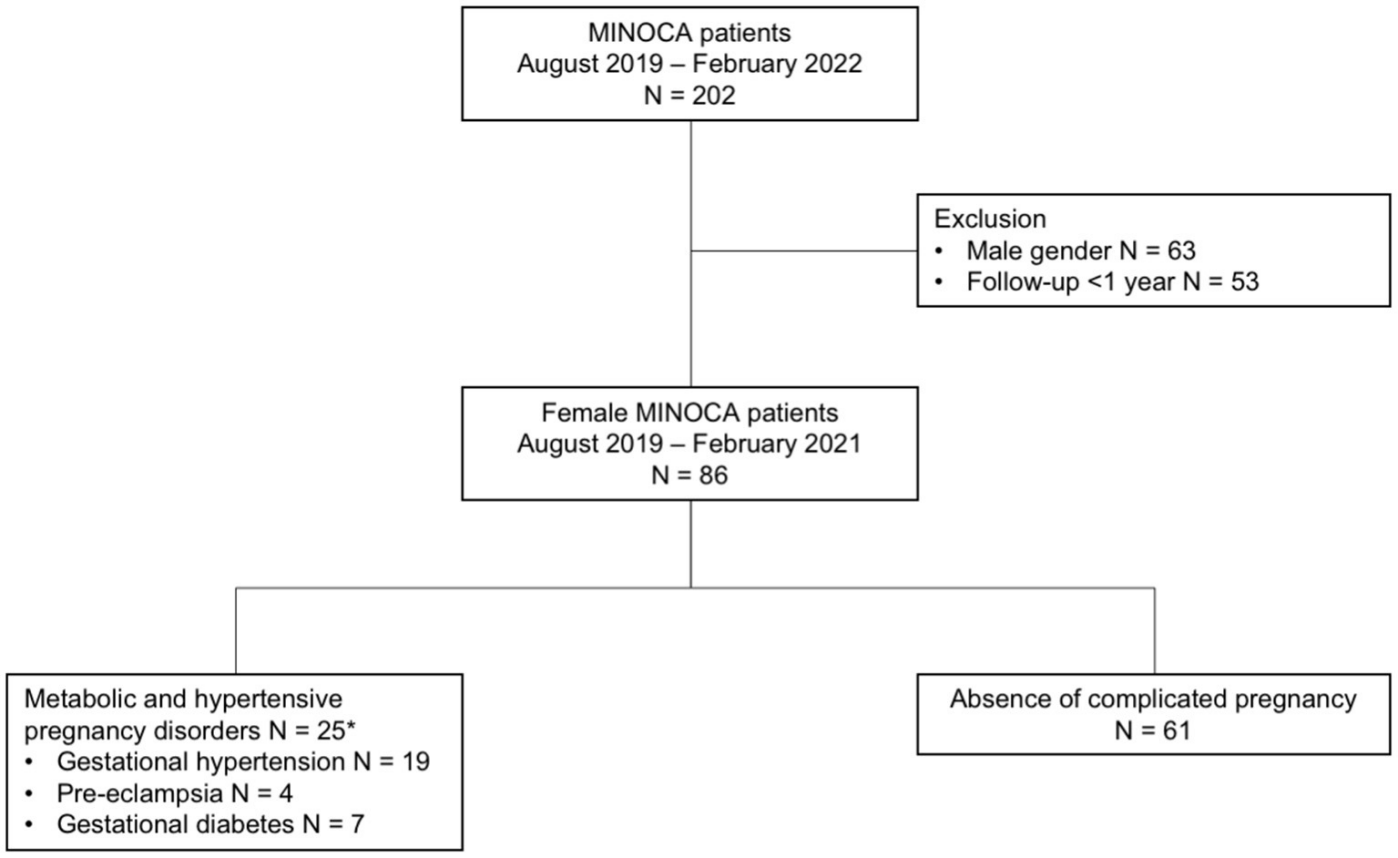
Flow-chart of included MINOCA patients. ^*^As some patients had multiple metabolic and hypertensive pregnancy disorders, numbers do not sum to 25.

From April 2019 to February 2022, a total of 202 MINOCA patients were enrolled in the MINOCA registry. After excluding patients without 1-year follow-up (*n* = 53), 86 female MINOCA patients remained eligible for analysis. Of them, 25 (29%) had a history of MHPD, including GH (*n* = 19; 22.1%), PE (*n* = 4; 4.7%), and GDM (*n* = 7; 8.1%; Figure 1).

### Patient Characteristics

In the overall study population, the mean age was 65.0 ± 11.1 years (similar across study groups), and the majority of patients were postmenopausal (88.1%). At the index hospitalization, systolic and diastolic blood pressure as well as cardiac biomarkers were similar across groups. Whilst three patients were in atrial fibrillation (3.5%), the remaining study participants were in sinus rhythm at the time of presentation at the cardiac emergency department (CED). Left bundle branch block was present in 6 (7.0%) and ST-segment deviation in 45 (52.3%) patients.

As shown in Table 2, the MHPD group showed higher rates of chronic hypertension (84.0 vs. 55.7%; *p* = 0.013), hypercholesterolemia (64.0 vs. 34.4%; *p* = 0.012), a family history of CVD (84.0 vs. 45.9%; *p* = 0.001), as well as gout or rheumatic arthritis (16.0 vs. 1.6%; *p* = 0.024) compared to NP patients. Contrastingly, NP patients were more often smokers (or former smokers; 78.3%) compared to MHPD (45.8%, *p* = 0.004) patients. Patients in the MHPD group were more likely to use antihypertensive medications at baseline, particularly angiotensin-converting enzyme inhibitors (ACE-i) or angiotensin receptor blockers (ARB; 56.0 vs. 27.9%; *p* = 0.014) and statins compared to the NP group, although the latter was borderline significant (44.0 vs 23.0%, *p* = 0.05). Use of oral anticoagulants, calcium channel blockers was equal in both groups (Table 2).

**Table 2:** Baseline characteristics.

	**Total**	**MHPD**	**NP**	***p*-Value**
	***n* = 86**	***n* = 25**	***n* = 61**	
**Mean age, years**	65.0 ± 11.1	66.5 ± 12.4	64.4 ± 10.5	0.42
**BMI, kg/m** ^**2**^	25.5 [22.3–28.5]	25.4 [22.5–28.7]	25.6 [22.2–28.5]	0.68
**Systolic blood pressure, mmHg**	152 [130–170]	153 [130–175]	150 [130–168]	0.72
**Diastolic blood pressure, mmHg**	85 [74–95]	83 [72–98]	85 [74–95]	0.71
**Heart rate at admission, bpm**	78 [70–86]	73 [66–87]	80 [71–87]	0.17
**Cardiovascular risk factors**
Current or previous smoker	58 (69.0)	11 (45.8)	47 (78.3)	0.004
Diabetes Mellitus	9 (10.5)	3 (12.0)	6 (9.8)	0.72
Chronic hypertension	55 (64.0)	21 (84.0)	34 (55.7)	0.013
Hypercholesterolemia	37 (43.0)	16 (64.0)	21 (34.4)	0.012
Previous AMI	8 (9.3)	2 (8.0)	6 (9.8)	1.00
Previous PCI	2 (8.0)	2 (8.0)	0 (−)	0.082
Previous stroke	11 (12.8)	4 (16.0)	7 (11.5)	0.72
Family history of CVD	49 (57.0)	21 (84.0)	28 (45.9)	0.001
Gout or rheumatic arthritis	5 (5.8)	4 (16.0)	1 (1.6)	0.024
Exercise <30 min/day	19 (22.4)	5 (20.0)	14 (23.3)	0.74
Emotional stress^*^	46 (54.1)	15 (60.0)	31 (51.7)	0.48
Peripheral artery disease	6 (7.1)	2 (8.0)	4 (6.7)	1.00
**Concomitant medical history**
Miscarriages	14 (18.7)	6 (24.0)	8 (16.0)	0.53
Postmenopausal status	74 (88.1)	21 (84.0)	53 (89.8)	0.48
COPD	15 (17.4)	1 (4.0)	14 (23.0)	0.057
Atrial fibrillation	6 (7.0)	3 (12.0)	3 (4.9)	0.35
Sleep apnea	2 (2.3)	0 (-)	2 (3.3)	1.00
**Medication use at home**
Aspirin	22 (25.6)	6 (24.0)	16 (26.2)	0.83
P2Y12-inhibitor	3 (3.5)	1 (4.0)	2 (3.3)	1.00
Oral anticoagulants	4 (4.7)	3 (12.0)	1 (1.6)	0.072
Betablocker	17 (19.8)	7 (28.0)	10 (16.4)	0.24
ACE-i/ARB	31 (36.0)	14 (56.0)	17 (27.9)	0.014
Calcium channel blocker	14 (16.3)	7 (28.0)	7 (11.5)	0.10
Statin	25 (29.1)	11 (44.0)	14 (23.0)	0.051

*Values are n (%), mean ± standard deviation or median [interquartile range]*.

* *Self-reported stress as assessed by the questionnaire at baseline*.

*ACE-i, angiotensin-converting enzyme inhibitor; AMI, acute myocardial infarction; ARB, angiotensin receptor blocker; BMI, body mass index; COPD, chronic obstructive pulmonary disease; CVD, cardiovascular disease; MHPD, metabolic and hypertensive pregnancy disorders; NP, normal pregnancy; PCI, percutaneous coronary intervention*.

### Coronary Angiography

Moderate atherosclerosis was present equally in the MHPD and NP group (73 vs. 75%; *p* = 0.48; Table 3). LV-angiography was performed in 16 (18.6%) patients, of whom eight were diagnosed with Takotsubo cardiomyopathy. No group differences were found with respect to coronary angiography findings. In total, four (4.7%) patients underwent intracoronary microvascular resistance testing, of whom only 1 showed increased microvascular resistance (CFR 2.4 and IMR 29). Of those four patients, two underwent additional spasm provocation testing. Both had inducible spasm by intracoronary acetylcholine infusion: one patient solely had epicardial spasm, while the other showed mixed microvascular dysfunction and spasm.

**Table 3:** Additional investigations.

	**Total**	**MHPD**	**NP**	***p*-Value**
	***n* = 86**	***n* = 25**	***n* = 61**	
**Laboratory findings**
Maximum hsTnT, ng/L	102 [49–315]	63 [43–199]	110 [51–365]	0.19
Maximum CK, U/L	145 [89–298]	123 [88–282]	158 [90–303]	0.57
NT-proBNP	152 [82–889]	227 [77–1102]	152 [77–1765]	0.70
Creatinine, μmol/L	70 [59–78]	70 [58–78]	69 [59–79]	0.79
LDL, mmol/L	2.8 ± 1.0	2.7 ± 1.0	2.9 ± 1.0	0.40
**Electrocardiography**
Sinus rhythm	83 (96.5)	24 (96.0)	59 (96.7)	1.00
Left bundle branch block	6 (7.0)	0 (–)	6 (9.8)	0.18
Right bundle branch block	1 (1.2)	0 (–)	1 (1.6)	1.00
ST-deviation	45 (52.3)	12 (48.0)	33 (54.1)	0.73
Dynamic during hospitalization	35 (40.7)	9 (36.0)	26 (42.)	0.57
**Echocardiography**	76 (88.4)	19 (76.0)	57 (93.4)	0.057
LVEF <45%	11 (15.3)	1 (5.3)	10 (18.9)	0.27
Wall motion disturbances	29 (38.7)	6 (31.6)	23 (41.1)	0.46
Interventricular septal thickness, cm	1.0 ± 1.1	0.8 ± 0.2	1.1 ± 1.3	0.13
Left ventricular posterior wall thickness, cm	0.8 ± 0.2	0.8 ± 0.2	0.8 ± 0.1	0.46
Deceleration time, ms	190 ± 42	170 ± 32	196 ± 44	0.11
E/A ratio	0.9 ± 0.3	0.9 ± 0.3	0.9 ± 0.3	0.88
E/e' septal	11.4 ± 4.1	12.3 ± 3.8	11.1 ± 4.2	0.35
E/e' lateral	9.5 ± 3.6	8.7 ± 3.9	9.8 ± 3.5	0.33
E/e' average	10.5 ± 3.5	10.6 ± 3.6	10.4 ± 3.5	0.86
Tricuspid regurgitation, m/s	2.6 ± 0.4	2.6 ± 0.5	2.6 ± 0.4	0.93
Right ventricular systolic pressure, mmHg	32 ± 10	33 ± 13	31 ± 8	0.66
**Invasive coronary angiography**
Moderate coronary atherosclerosis	63 (73.3)	17 (68.0)	46 (74.2)	0.48
Left ventricular angiography	16 (18.6)	2 (8.0)	14 (23.0)	0.14
Takotsubo	8 (50.0)	–	8 (57.1)	
OCT	3 (3.5)	0 (–)	3 (4.9)	0.55
Abnormal	–	–	–	
Intracoronary provocation testing	2 (2.3)	1 (4.0)	1 (1.6)	0.50
Abnormal	2 (100)	1 (100)	1 (100)	
Intracoronary resistance testing	4 (9.5)	1 (9.1)	3 (9.7)	1.00
Abnormal	1 (25.0)	1 (100)	–	
**Cardiac magnetic resonance imaging**	32 (37.2)	9 (36.0)	23 (37.7)	0.88
LVEF, %	58 ± 9	59 ± 6	57 ± 11	0.58
End-diastolic volume, ml	133 ± 28	130 ± 29	134 ± 28	0.72
End-systolic volume, ml	56 ± 17	53 ± 13	57 ± 19	0.62
Left ventricular mass, g	92 ± 21	85 ± 18	94 ± 22	0.32
LGE present, % of performed CMR	12 (37.5)	1 (11.1)	11 (47.8)	0.054
LGE distribution, % of patients with LGE
Ischemic (subendocardial/transmural)	7 (58.3)	–	7 (63.6)	
Nonischemic (midmyocardial/(sub)epicardial)	3 (25.0)	1 (100)	2 (18.2)	
Mixed	2 (16.7)	–	2 (18.2)	
**Additional coronary investigations**
CT-angiography	23 (26.7)	5 (20.0)	18 (29.5)	0.002

*Values are n (%), mean ± standard deviation or median [interquartile range]*.

*CMR, cardiac magnetic resonance imaging; CK, creatinine kinase; CT, computed tomography; IVUS, intravascular ultrasound; LDL, low-density lipoprotein; LGE, late gadolinium enhancement; MHPD, metabolic and hypertensive pregnancy disorders; NP, normal pregnancy; NT-proBNP, N-terminal pro-brain natriuretic peptide; OCT, optical coherence tomography*.

Three (3.5%) patients underwent OCT revealing mild atherosclerosis, but no presence of plaque rupture, plaque erosion, coronary thrombus or coronary dissection.

### Echocardiography

In total, 76 patients (88.4%) underwent echocardiography during index hospitalization. Reduced LVEF <45% was equally present in the MHPD and NP group (5.3 vs. 18.9%; *p* = 0.27). No significant differences in systolic or diastolic parameters were observed across groups (Table 3).

### Cardiac Magnetic Resonance Imaging

Cardiac magnetic resonance (CMR) was performed in 32 (37.2%) patients at a median of 3434 [10-79] days after the index coronary angiography. Mean LVEF on CMR was 58 ± 9% and comparable between both groups (59 vs. 57%; *p* = 0.58). No differences in end-diastolic volume, end-systolic volume, or left ventricular mass was found. LGE abnormalities were found less frequently in the MHPD group, though borderline significant (11.1 vs. 47.8%; *p* = 0.054). An ischemic (subendocardial, or transmural) pattern was identified in most patients (7/12), followed by a nonischemic (sub)epicardial and midmyocardial pattern (3/12 patients), and lastly a mixed LGE pattern (2/12 patients; Table 3). In another four patients (all from the NP group) a form of cardiomyopathy was found, which were two Takotsubo cardiomyopathies and two dilated cardiomyopathies. The remaining 16 patients showed normal findings on CMR.

### MINOCA Diagnosis

A trend toward no specific cause found for the MINOCA event was observed in MHPD patients compared to the NP group (64.0 vs. 42.6%, *p* = 0.072). All other related diagnoses where equally present in the MHPD and NP groups, including a coronary related diagnosis (16.0 vs. 26.2%, *p* = 0.31 respectively), myocarditis (8.0 vs. 3.0%, *p* = 0.58), cardiomyopathy (including Takotsubo and dilated cardiomyopathy (8.0 vs. 18.0%, *p* = 0.33 respectively), or other miscellaneous causes (e.g., cardiac arrhythmias or pulmonary embolism) (4.0 vs 9.8%; *p* = 0.37; Table 4).

**Table 4:** Final diagnosis of MINOCA.

	**Total**	**MHPD**	**NP**	***p*-Value**
	***n* = 86**	***n* = 25**	***n* = 61**	
**Final diagnosis**
Coronary related^*^	20/86 (23.3)	4/25 (16.0)	16/61 (26.2)	0.31
Plaque rupture	8/20 (40.0)	2/5 (50.0)	6/16 (37.5)	
Coronary embolism	3/20 (15.0)	1/4 (25.0)	2/16 (12.5)	
Coronary spasm	6/20 (30.0)	1/4 (25.0)	5/16 (31.3)	
Spontaneous coronary artery dissection	3/20 (15.0)	–	3/16 (18.8)	
Microvascular dysfunction	1/20 (5.0)	1/4 (25.0)	–	
*Myocarditis*	4/86 (4.7)	2/25 (8.0)	2/61 (3.3)	0.58
*Cardiomyopathy^‡^*	13/86 (15.1)	2/25 (8.0)	11/61 (18.0)	0.33
Takotsubo	11/13 (84.6)	2/2 (100)	9/11 (81.8)	
Dilated cardiomyopathy	2/13 (15.4)	–	2/11 (18.2)	
*MINOCA undefined*	42/86 (48.8)	16/25 (64.0)	26/61 (42.6)	0.072
*Other*	7/86 (8.1)	1/25 (4.0)	6/61 (9.8)	0.37

*Values are n (%)*.

* *Includes plaque rupture, coronary embolism, coronary spasm, microvascular dysfunction and spontaneous coronary artery dissection. As 1 patient had mixed microvascular dysfunction and spasm, numbers do not sum to 100%*.

‡ *Includes Takotsubo cardiomyopathy and dilated cardiomyopathy*.

*MINOCA, myocardial infarction with non-obstructive coronary arteries; MHPD, metabolic and hypertensive pregnancy disorders; NP, normal pregnancy*.

## Discussion

We performed an observational cohort study among female MINOCA patients with and without prior obstetric complications. First, approximately one-third of these MINOCA patients suffered prior obstetric complications, highlighting the importance of obtaining an obstetric history to uncover potential CVD risk factors. Accordingly, physicians should aim to aggressively manage modifiable cardiovascular risk factors through early interventions in this vulnerable patient population to ultimately mitigate the negative impact on cardiovascular outcomes. Second, the group of women with a history of MHPD showed a higher prevalence of conventional cardiovascular risk factors, including hypertension, hypercholesterolemia, a family history of CVD, and the presence of gout or rheumatic disorders. Moreover, in two-thirds of these patients, no specific cause for the MINOCA event was found, which occurred 20% more often compared to the NP group. However, this did not attain statistical significance. A lack of a clear underlying specific cause may adversely affect long term outcomes due to possibly insufficient medical treatment, or, even worse, no treatment at all in this particular subset of patients.

The prevalence of GH and PE was 22.1 and 4.7% in our study population, which is in particular for GH considerably higher than in the general population with prior pregnancies. Oliver-Williams et al. (14) performed a retrospective cohort study among over two million women from England with over four million live births during 1997–2015, reporting a prevalence of 4.8 and 3.9% for GH and PE respectively, while Arnaout et al. (20) report an even lower prevalence of GH and PE, 3.0 and 3.7% respectively. In the latter study, GDM was observed in 6.5% of 1.6 million pregnancies in California, which is lower compared to the current study cohort, namely 8.1%. As we used self-report questionnaires in elderly women to assess the history of obstetric complications, the prevalence might have been underestimated as the definition of and communication with regards to obstetric complications may not have = been as accurate and clearly defined in the past.

On the other hand, the current finding of almost one-third of females with MINOCA to have had prior obstetric complications is in line with previous research in other cohorts of AMI patients. Grand'Maison et al. (21) reported a combined prevalence of GH, PE and GDM of 38% among a general AMI population including both patients with obstructive and non-obstructive coronary artery disease. The VIRGO study also showed high rates of PE, GDM, and miscarriages in up to 30%, which were equally distributed across MINOCA as well as AMI with obstructive coronary artery disease (10).

Previous studies established strong correlations between hypertensive disorders of pregnancy and myocardial infarction, heart failure, and stroke. In contrast, merely modest associations were found between GDM and adverse cardiovascular outcomes (20). A recent large cohort study revealed that hypertensive disorders of pregnancy were independently associated with atherosclerotic cardiovascular disease, despite adjustment for traditional CVD risk factors (22). Even among a relatively small cohort of women with a confirmed cardiac ischemic event, we found that patients with a history of metabolic and hypertensive pregnancy disorders had a high burden of CVD risk factors. Due to the relatively small sample size of our study, assessment of cardiovascular events such as recurrent myocardial infarction, stroke or cardiac death may not be reliable. In our study, a relative overrepresentation of conventional CVD risk factors in the MHPD group was found. This is in line with the fact that these patients were more commonly using cardiovascular medications at baseline, including ACE-i/ARBs, statins, and oral anticoagulants. Despite the pharmacologic treatment for CVD risk factors, MINOCA events still occurred. Nevertheless, ACE-i/ARBs and statins have shown to have beneficial effects on future adverse events in MINOCA patients (23).

In the past decade, an increasing number of studies focused on obstetric risk factors and CVD risk postpartum. Hypertensive disorders of pregnancy are known predictors of chronic hypertension, insulin resistance, and dyslipidemia in later stages of life (24–26). These obstetric risk factors may co-exist with other CVD risk factors, translating into an increased risk of developing cardiovascular events. Our study emphasizes the role of chronic hypertension as a key risk factor for CVD in this population since over 80% of MHPD patients were known to have chronic hypertension. Moreover, MHPD patients showed to have much higher rates of dyslipidemia, gout and rheumatic disorders, as well as a family history of CVD.

The mechanism responsible for chronic hypertension and adverse cardiovascular outcomes in women with prior GH and PE is considered complex and multifactorial. Proposed mechanisms refer to the role of the development of metabolic syndrome comprising abdominal obesity, insulin resistance, dyslipidemia, and hypertension (27). Another potential mechanism catalyzing development of the metabolic syndrome is the persistent enhanced inflammatory state following PE. Chronic systemic as well as local placental inflammation accelerates endothelial dysfunction, inciting the process of atherosclerosis, contributing to future coronary events (28, 29). In previous studies, elevated inflammatory markers were demonstrated not only in AMI patients with obstructive coronary artery disease, but also in MINOCA patients, emphasizing the potential complex interplay between hyperglycemia, inflammation and myocardial infarction (30). In addition, regardless of concomitant diabetes mellitus diagnosis, an elevated hyperglycemia in MINOCA at admission was shown to be an important predictor for adverse short and long-term clinical outcomes (31).

Hypertension is a strong risk factor for CVD. The cumulative effect of prolonged exposure to high blood pressure results in subclinical low-grade inflammation, endothelial dysfunction, and progressively irreversible changes in cardiac- and vascular structure and —function, increasing the risk of overt clinical cardiovascular events (32, 33). When diagnosed before irreversible cardiovascular dysfunction occurs, blood pressure can be modified by lifestyle adjustments or medication in an effort to prevent these events (34–39). Therefore, timely detection of hypertension is of utmost importance, particularly in formerly preeclamptic individuals, who are at two- to seven-fold increased risk for future CVD (40). Current guidelines recommend counseling and follow-up for CVD risk modification after preeclampsia, although hampered by the lack of prediction tools (41).

### Strength and Limitations

This study is the first observational cohort study shedding light on the relationship between MINOCA and obstetric history with a comprehensive cardiovascular assessment. Nevertheless, several limitations should be considered when interpreting the current study results. First, the relatively small study sample could potentially affect the ability to detect group differences, particularly in severe CVD events which occur at relatively low rates. Second, classification of obstetric complications was self-reported, and therefore some degree of patient-related misinterpretation or underreporting cannot be eliminated.

Third, although additional examination was performed in the majority of patients, one-third did not undergo additional diagnostic testing (other than routine coronary angiography and echocardiography). Only one-third of the study population underwent CMR, whilst current guidelines advocate this as a class IB recommendation in MINOCA cases without an obvious underlying cause (42). In addition, the median time to CMR was ~1 month which may have led to an overestimation of normal findings due to the transient nature of myocardial edema. This probably contributed to the large proportion of patients not given a definitive diagnosis, since CMR provided additional information with regards to the underlying diagnosis in half of patients (16/32).

This subgroup of patients might benefit from additional diagnostic measures in order to reveal or verify the underlying disease process, which in turn, might positively contribute toward optimal medical treatment and risk modification.

Fourth, due to the study design and the time span between pregnancy and the occurrence of the MINOCA event, it was not possible to determine whether patients were diagnosed with with hypertension or diabetes mellitus prior to their (first) pregnancy. This might have introduced a unknown degree of selection bias.

## Conclusion

Metabolic and hypertensive pregnancy disorders occur in almost one-third of female MINOCA patients. MINOCA patients with prior obstetric complications showed higher rates of conventional CVD factors compared to those without obstetric complications, in spite of pharmacologic treatment being more common. Moreover, patients with obstetric complications remained without a definitive diagnosis for the MINOCA event more often.

## Perspectives

Future long-term prospective studies that monitor women with previous obstetric complications after AMI are warranted to enhance our understanding of the risk of major adverse cardiovascular events. Moreover, clinical trials should aim to aggressively manage modifiable cardiovascular risk factors through early interventions in this vulnerable patient population to mitigate the negative impact on cardiovascular outcomes.

## Data Availability Statement

The raw data supporting the conclusions of this article will be made available by the authors, without undue reservation.

## Ethics Statement

The studies involving human participants were reviewed and approved by Medisch Ethische Toetsingscommissie Zuyderland. The patients/participants provided their written informed consent to participate in this study.

## Author Contributions

TP: conceptualization, formal analysis, investigation, methodology, validation, visualization, writing—original draft, and writing—review and editing. NV: investigation, methodology, validation, visualization, writing—original draft, and writing—review and editing. GJ, PW, MSt, LH, and CG: conceptualization, writing—original draft, and writing—review and editing. BK and MSp: conceptualization, supervision, and writing—review and editing. SR and AH: conceptualization, supervision, writing—original draft, and writing—review and editing. All authors contributed to the article and approved the submitted version.

## Conflict of Interest

The authors declare that the research was conducted in the absence of any commercial or financial relationships that could be construed as a potential conflict of interest.

## Publisher's Note

All claims expressed in this article are solely those of the authors and do not necessarily represent those of their affiliated organizations, or those of the publisher, the editors and the reviewers. Any product that may be evaluated in this article, or claim that may be made by its manufacturer, is not guaranteed or endorsed by the publisher.
